# Benchmarking tools for a priori identifiability analysis

**DOI:** 10.1093/bioinformatics/btad065

**Published:** 2023-01-31

**Authors:** Xabier Rey Barreiro, Alejandro F Villaverde

**Affiliations:** Department of Systems and Control Engineering, Universidade de Vigo, 36310 Vigo, Galicia, Spain; Department of Systems and Control Engineering, Universidade de Vigo, 36310 Vigo, Galicia, Spain; CITMAga, 15782 Santiago de Compostela, Galicia, Spain

## Abstract

**Motivation:**

The theoretical possibility of determining the state and parameters of a dynamic model by measuring its outputs is given by its structural identifiability and its observability. These properties should be analysed before attempting to calibrate a model, but their a priori analysis can be challenging, requiring symbolic calculations that often have a high computational cost. In recent years, a number of software tools have been developed for this task, mostly in the systems biology community. These tools have vastly different features and capabilities, and a critical assessment of their performance is still lacking.

**Results:**

Here, we present a comprehensive study of the computational resources available for analysing structural identifiability. We consider 13 software tools developed in 7 programming languages and evaluate their performance using a set of 25 case studies created from 21 models. Our results reveal their strengths and weaknesses, provide guidelines for choosing the most appropriate tool for a given problem and highlight opportunities for future developments.

**Availability and implementation:**

https://github.com/Xabo-RB/Benchmarking_files.

## 1 Introduction

Systems biology models are often given by nonlinear ordinary differential equations (ODEs) with unknown parameters, which must be estimated by fitting the model to experimental data. This task, known as model calibration (Villaverde *et al.*, 2022), can only be performed successfully if the model is identifiable. Unidentifiability may lead to inaccurate inferences of mechanistically meaningful parameters, as well as to the inability to make correct predictions of certain variables (Eisenberg and Jain, 2017; Janzén *et al.*, 2016; Muñoz-Tamayo *et al.*, 2018). It is common to distinguish between structural and practical identifiability (Wieland *et al.*, 2021). Structural identifiability is a theoretical property that is fully determined by the model equations, that is, it depends on the system dynamics, the measurable outputs and the admissible inputs. It is also called a priori identifiability, since it can be tested before performing experiments and collecting data. Some authors consider a priori identifiability as a particular type of structural identifiability (Wieland *et al.*, 2021), while others use both terms interchangeably (Anstett-Collin *et al.*, 2020); in this article, we adopt the latter terminology. A related property is observability, which is the possibility of inferring the internal state of a system from observations of its outputs. By considering model parameters as constant state variables, a priori identifiability can be recast as a particular case of observability. Structural unidentifiabilities are determined by the model equations and can be caused by Lie symmetries (Anguelova *et al.*, 2012; Merkt *et al.*, 2015). Removing them entails modifying the model equations, for example by reparameterizing the ODEs or by enlarging the output function. A further distinction can be made between structural *local* identifiability (SLI) and structural *global* identifiability (SGI). A parameter that has the SLI property can be uniquely inferred in a neighbourhood of its nominal value, but a finite number of indistinguishable solutions may exist in the parameter space. In contrast, a parameter with the SGI property has a unique solution in the whole parameter space.

The a priori analysis of identifiability and observability is mathematically involved, requiring symbolic computations that quickly become computationally expensive even for models of moderate size. Hence, a number of methodologies and software tools have been developed for its study. Two papers provided an overview of the state of the art in 2011: Miao *et al.* (2011) reviewed the theoretical foundations of practical and structural identifiability analysis methods, and Chis *et al.* (2011) performed a computational comparison of structural identifiability algorithms. At that time, only two software toolboxes were publicly available for this task: DAISY (Bellu *et al.*, 2007) and GenSSI (Chiş *et al.*, 2011); hence, Chis *et al.* implemented a number of other approaches themselves. Raue *et al.* (2014) compared DAISY with two identifiability analysis tools that had been recently presented: the Exact Arithmetic Rank (EAR) (Karlsson *et al.*, 2012) and the Profile Likelihood (PL), a technique for *a posteriori* analysis (Raue *et al.*, 2009).

Since the publication of Raue *et al.* (2014), a significant number of software tools for structural identifiability analysis have been presented, including the web app COMBOS (Meshkat *et al.*, 2014), the Matlab toolboxes STRIKE-GOLDD (Villaverde *et al.*, 2016), GenSSI2 (Ligon *et al.*, 2018), ORC-DF (Maes *et al.*, 2019) and rational ORC-DF or RORC-DF (Shi and Chatzis, 2022), the Maple toolboxes SIAN (Hong *et al.*, 2019) and ObservabilityTest (Sedoglavic, 2002), the Julia packages StructuralIdentifiability (Dong *et al.*, 2022) and SIAN (https://github.com/alexeyovchinnikov/SIAN-Julia) and the Python tool StrikePy (Rey Rostro and Villaverde, 2022). However, an assessment of their relative strengths and weaknesses is currently lacking. Given their different theoretical foundations, capabilities and computational performances, there is a clear need for their critical analysis and comparison. Some results in this direction were presented in Hong *et al.* (2019), where the performance of four tools for SGI analysis (DAISY, COMBOS, GenSSI and SIAN) was compared using six case studies.

In this article, we address this need by performing a thorough comparison of the software tools currently available for analysing structural identifiability and observability. We consider symbolic computation methods that perform the said analyses a priori. We do not consider numerical approaches, such as the aforementioned PL (Raue *et al.*, 2009) or sensitivity-based methods (Stigter and Joubert, 2021; Stigter and Molenaar, 2015), which perform *a posteriori* analyses and can complement the techniques reviewed here (Wieland *et al.*, 2021). Thus, we consider thirteen different tools, available in seven different environments: Matlab, Maple, Mathematica, Julia, Python, Reduce and web-based applications. We benchmark their performance using a total of 25 variants of 21 basic models, of different sizes and complexities, taken from the systems biology literature. We discuss the strengths and weaknesses of each tool, and provide guidelines for choosing the most appropriate tool for a given problem.

## 2 Materials and methods

### 2.1 Structural identifiability and observability concepts

We consider dynamic models described by ordinary differential equations in state space form:

(1) $$\begin{array}{c} \Sigma =\{\begin{array}{c} \dot{x}=f(t,x(t),u(t),\theta ,w(t)),\\ y(t)=h(x(t),u(t),\theta ,w(t)),\\ x(0)=x^{0}(\theta ) \end{array} \end{array}$$

where $x(t)\in R^{n}$ is a vector of state variables, $y(t)\in R^{m}$ is a vector of outputs or measurements, $u(t)\in R^{q}$ is the vector of known inputs, $w(t)\in R^{q_{w}}$ is the vector of unknown inputs, and $\theta \in R^{p}$ is the unknown parameter vector. Initial conditions may be functions of unknown parameters, or generic unknown values. We write individual parameters and state variables with subindices (i.e. $\theta_{i}$, $x_{i}$), and we denote as $y(t,u(t),\theta^{*})$ the output of a model Σ for a specific parameter vector $\theta^{*}$ and input *u*(*t*).

### 2.2 Definitions

Many definitions of a priori identifiability can be found in the literature. They describe similar properties with subtle differences among them. For a detailed account of said definitions and their nuances, we refer the interested reader to Anstett-Collin *et al.* (2020). In what follows, we provide only brief descriptions of these concepts, which we attempt to keep as simple as possible. Roughly speaking, a dynamic model is *observable* if its current state vector x(t) can be determined from knowledge of the future values of the output y(t) and input functions u(t) in finite time. Likewise, it is *identifiable* if its parameter vector θ can be determined from the output y(t) and input functions u(t) in finite time. It is common to distinguish between local and global identifiability.

Definition 2.1 .

*Structural Local Identifiability: a parameter* $\theta_{i}$*of a dynamic model* Σ*is structurally locally identifiable (SLI) if, for almost all possible parameter vectors, admissible inputs and initial conditions, there is a neighbourhood* $N(\theta^{*})$*in which the equality* $y(t,u(t),\tilde{\theta })=y(t,u(t),\theta^{*})$*holds if and only if* ${\tilde{\theta }}_{i}=\theta_{i}^{*}$.

Definition 2.2 .

*Structural Global Identifiability: a parameter* $\theta_{i}$*of a dynamic model* Σ*is structurally globally identifiable (SGI) if, for almost all possible parameter vectors, admissible inputs and initial conditions, the equality* $y(t,u(t),\tilde{\theta })=y(t,u(t),\theta^{*})$*holds if and only if* ${\tilde{\theta }}_{i}=\theta_{i}^{*}$.

Note that SGI parameters are also SLI. If the above conditions do not hold, the parameter is *structurally unidentifiable* (SU). A model is said to be SGI (respectively, SLI) if all its parameters are SGI (resp., at least SLI). If it has at least one SU parameter, the model is called SU. Likewise, we could provide local and global definitions of observability. Nevertheless, the observability of nonlinear systems was originally defined in a differential geometric framework as a local property, and it is therefore common to consider observability only from a local point of view:

Definition 2.3 .

*Observability: a state variable* $x_{i}(\tau )$*is observable if, for almost all possible parameter vectors and almost all initial conditions, there is a neighbourhood* $N(\theta^{*})$*in which the equality* $y(t,\tilde{x}(\tau ))=y(t,x^{*}(\tau ))$*holds if and only if* ${\tilde{x}}_{i}(\tau )=x_{i}^{*}(\tau )$.

### 2.3 The differential geometry approach

Structural *local* identifiability can be analysed with a differential geometric approach that checks the Observability Rank Condition (ORC). The ORC determines *local weak observability* (Hermann and Krener, 1977):

Definition 2.4 .

*Local weak observability: Let U be an open subset in* $R^{n}$*, and let indistinguishability be an equivalence relation on* $R^{n}$*. We denote as* $I(x_{0},U)$*all points* $x_{i}\in U$*that are indistinguishable from* $x_{0}$*. The system* Σ*is locally weakly observable at* $x_{0}$*if* $I(x_{0},V)=x_{0}$*for every open neighbourhood V of* $x_{0}$*contained in U.*

A model is locally weakly observable if it is possible to distinguish each state vector from its neighbours. Local weak observability can also be applied to parameters by considering them as constant state variables. In this view, an SLI parameter is a weakly locally observable state, and this approach can be used to test whether a parameter is SLI.

Before defining the ORC we need a few more mathematical preliminaries. Let $L_{v}(\phi )(x):=<d\phi ,v>$ denote the differentiation of an infinitely differentiable function ϕ on $R^{n}$ by a vector field *v* on $R^{n}$, where dϕ is the gradient of ϕ and <> the scalar product. We denote by Φ(t,x) the flow of a vector field *v* on $R^{n}$. The Taylor series of ϕ(Φ(t,x)) with respect to *t* are called Lie series, and they are given by:

$$\begin{array}{c} \phi (\Phi (t,x))=\sum_{k=0}^{\infty }\frac{t^{k}}{k!}L_{v}^{k}(\phi )(x) \end{array}.$$

Let ϱ denote the space spanned by $L_{f}^{q}h_{i}$ at $x_{0}$ for q≥0 and i=1,..,m, for all vector fields f(x,u). The space spanned by the gradients of the elements of ϱ is defined by $d\varrho ={\text{span}}_{R_{x}}\{d\phi :\;\;\;\;\;\;\phi \in \varrho \}$, where $R_{x}$ indicates the field of meromorphic functions on $R^{n}$. We call dϱ the observability matrix, O(x). Thus, the observability—and therefore the structural local identifiability—of a model can be tested with the following theorem:

Theorem 2.1 .

*Observability Rank Condition (ORC) (Hermann and Krener, 1977): if the system* Σ*(1) satisfies* $\mathrm{rank}(O(x_{0}))=n$*, then it is locally weakly observable around* $x_{0}$.

### 2.4 The differential algebra approach

Structural *global* identifiability can be tested with a differential algebra approach. It relies on finding algebraic equations that relate the model parameters with the inputs and outputs (Ljung and Glad, 1994). The definition of algebraic identifiability has been shown to be essentially equivalent to our definition of global structural identifiability (Hong *et al.*, 2020). Importantly, this approach introduces a restriction on the class of systems that can be analysed: instead of being applicable to general non-linear ODE systems of the form (1), it requires that the ODE functions are rational. The same restriction is shared by other methods, as will be detailed in Section 2.5.

Differential algebra methods replace Equation (1) of the system Σ by a set of polynomial differential equations that depend only on (y,u), i.e. they rewrite Σ in implicit form (Saccomani *et al.*, 2001). These input–output equations preserve the dynamics of the model output while eliminating the state variables from the equations and can be obtained in different ways. The resulting functions constitute the *exhaustive summary* of the model (Walter and Lecourtier, 1982). A vector c(θ) is an exhaustive summary of a model if it only contains the information about θ that can be inferred from u(t) and y(t). Checking the injectivity of the map c(θ) amounts to assessing the identifiability of the model.

### 2.5 Overview of tools for analysing structural identifiability and observability a priori

In the remainder of this section, we provide brief conceptual descriptions of those symbolic methods that have publicly available software implementations. They are listed in Table 1. Then, we evaluate their computational performance in Section 3.

**Table 1.** Software tools evaluated in this work

			Features							
Tool	Reference	Language	Global	Symmetries	ICs	Unknown in	Reparamet	Nonrational	Combin	# solutions
ObservabilityTest	Sedoglavic (2002)	Maple		✓			✓			
EAR	Karlsson *et al.* (2012)	Mathematica		✓	✓		✓			
STRIKE-GOLDD (FISPO)	Villaverde *et al.* (2019)	Matlab		✓		✓	✓	✓		
STRIKE-GOLDD (ProbObsTest)	Díaz *et al.* (2023)	Matlab		✓		✓	✓			
StrikePy	Rey Rostro and Villaverde (2022)	Python				✓				
RORC-DF	Shi and Chatzis (2022)	Matlab				✓				
GenSSI2	Ligon *et al.* (2018)	Matlab	✓		✓			✓		
SIAN v1.5 (Maple)	Hong *et al.* (2019)	Maple	✓							✓
SIAN v1.1.1 (Julia)		Julia	✓							✓
SIAN (WebApp)	Ilmer *et al.* (2021)	Maple (web app)	✓						✓	✓
DAISY	Bellu *et al.* (2007)	Reduce	✓		✓					✓
COMBOS	Meshkat *et al.* (2014)	Maxima (web app)	✓		✓				✓	✓
Structural-Identifiability v0.3.0	Dong *et al.* (2022)	Julia	✓						✓	

*Note*: All tools are in principle capable of testing for local identifiability. The ‘Features’ columns indicate which methods are capable of the following tasks: analysing global identifiability (‘Global’), finding the Lie symmetries in the model equations (‘Symmetries’), testing for specific initial conditions (‘ICs’), considering models with unknown inputs (‘Unknown in’), finding identifiable model reparameterizations (‘Reparamet’), analysing non-rational models (‘Nonrational’), finding identifiable parameter combinations (‘Combin’) and calculating the number of solutions (‘# solutions’). A tick symbol indicates that the method possesses the corresponding feature.

The tools considered in this work can be classified into two broad classes, depending on their approach (differential geometry or differential algebra), although some of them have elements of both—e.g. the generating series approach implemented in GenSSI. Furthermore, not all tools provide the same features. For example, some methods—in fact, most of them—are only applicable to rational models. Likewise, some algorithms allow the definition of specific initial conditions, while others do not. Another difference concerns the possibility of considering models with unknown inputs. Finally, some software tools go beyond structural identifiability and observability analysis, informing about the existence of symmetries, identifiable parameter combinations or model reparameterizations.

#### 2.5.1 DAISY

DAISY (Differential Algebra for Identifiability of SYstems) was the first symbolic computation tool presented for SGI analysis (Bellu *et al.*, 2007). It is a differential algebra software written in REDUCE version 3.8, a free symbolic language. Its algorithm writes the input–output relation of the system in implicit form, i.e. as a set of *m* polynomial differential equations in the variables (y,u), eliminating the dependence on *x*. After ranking the model variables, the characteristic set of the differential ideal is computed with Ritt’s pseudodivision algorithm (Ritt, 1950). This yields differential equations whose coefficients depend on the parameter vector θ. Each of the equations is normalized, making it monic. The family of new functions is the exhaustive summary c(θ), which encapsulates the parameter dependence of the output and whose injectivity has to be checked. DAISY solves the system of algebraic nonlinear equations c(θ) with the algorithm by Buchberger and Winkler (1998), which calculates the Gröbner basis of the system and provides the number of solutions for each parameter.

#### 2.5.2 COMBOS

COMBOS is a web-based application (Meshkat *et al.*, 2014) for SGI analysis that uses the computer algebra system Maxima. It uses a differential elimination method as an alternative to Ritt’s pseudodivision algorithm. COMBOS extends the capabilities of DAISY by providing as additional information the simplest globally identifiable combinations of the unidentifiable parameters. For locally identifiable parameters, COMBOS determines the maximum number of local solutions. DAISY and COMBOS differ in the way in which they handle initial conditions. A model that is in principle identifiable for generic initial conditions might be unidentifiable for certain initial conditions from which it is not accessible (Saccomani *et al.*, 2003). If we provide specific initial conditions, the results of both software tools are consistent if the system is accessible from those initial conditions, but they may differ in case of inaccessibility. This is because COMBOS, unlike DAISY, does not consider all possible inaccessible cases.

#### 2.5.3 SIAN

SIAN (Structural Identifiability ANalyser) is an open-source software tool for SGI analysis. It combines differential algebra methods with the Taylor series approach (Hong *et al.*, 2019, 2020). SIAN creates a map that binds the parameter values and initial conditions to output functions. By replacing the latter with truncations of their Taylor series, the map is reduced to another map between finite-dimensional spaces. To this end, SIAN determines the order of truncation that contains enough information for the identifiability analysis. The result is correct with a given probability, which is estimated within the algorithm. SIAN is available as Maple code, as Julia code and as a web app in the Maple Cloud server (Ilmer *et al.*, 2021). We have tested the three tools separately since each one has different capabilities and features; notably, the web app can compute identifiable combinations using an algorithm from Ovchinnikov *et al.* (2021).

#### 2.5.4 StructuralIdentifiability

StructuralIdentifiability.jl (Dong *et al.*, 2022) is the most recent tool for analysing SGI. It is a package implemented in the Julia language as a part of SciML ecosystem, an open source software for scientific machine learning. It follows a differential algebra approach, computing the input–output equations via projections to improve the performance. Subsequently, it performs the injectivity test, which, similarly to DAISY and COMBOS, is computed in a probabilistic way; unlike them, however, StructuralIdentifiability guarantees the correctness of the result with a given probability.

#### 2.5.5 GenSSI

GenSSI (Generating Series for testing Structural Identifiability) is a Matlab toolbox for SGI analysis. It was originally presented in Chiş *et al.* (2011), and a substantially new implementation (GenSSI 2.0) appeared in Ligon *et al.* (2018). It combines the generation series approach with identifiability tableaus. The generating series approach resembles the power series expansion (Pohjanpalo, 1978), which is based on the idea that the Taylor series expansions of the output functions include all the relevant information for analysing identifiability. By computing symbolically the successive Lie derivatives of the output functions with respect to parameters and states, an exhaustive summary is obtained, and from its injectivity the parameter identifiability can be established. Identifiability tableaus (Balsa-Canto *et al.*, 2010) are used for determining the number of solutions visually, helping to classify a parameter as SLI or SGI.

#### 2.5.6 ObservabilityTest

ObservabilityTest is a Maple tool for analysing SLI of rational models. It implements the probabilistic algorithm presented by Sedoglavic (2002), which evaluates the ORC efficiently, i.e. in bounded polynomial time. It achieves this goal by avoiding the symbolic computation of Lie derivatives when building the observability matrix, calculating instead the first terms of a power series expansion, specializing the variables on random integers and applying modular operations. When the model is unobservable, the power series approach searches for the Lie symmetries that cause the unobservability. Since it is the fastest algorithm for assessing SLI in rational models, it has been re-implemented in StructuralIdentifiability.jl (Julia), EAR (Mathematica) and STRIKE-GOLDD (Matlab).

#### 2.5.7 EAR

The Exact Arithmetic Rank (EAR), also known as IdentifiabilityAnalysis, is a Mathematica tool for SLI analysis (Karlsson *et al.*, 2012). It extends the probabilistic semi-numerical algorithm introduced by Sedoglavic (2002), which was later implemented in the Maple tool ObservabilityTest. As an enhancement over ObservabilityTest, EAR can consider either generic initial conditions (using the ‘observability analysis’ function) or initial conditions specialized to some numerical value (using the ‘identifiability analysis’ function). Furthermore, EAR can find certain Lie point symmetries in the model, and to compute the minimal output sets for achieving identifiability (Anguelova *et al.*, 2012).

#### 2.5.8 STRIKE-GOLDD

STRIKE-GOLDD (STRuctural Identifiability taKen as Extended-Generalized Observability with Lie Derivatives and Decomposition) is a Matlab toolbox that uses the differential geometry approach (Villaverde *et al.*, 2016). Besides analysing SLI, it can search for Lie symmetries and for identifiable reparameterizations (Massonis *et al.*, 2021). It implements three algorithms (i) *FISPO*, which is the most generally applicable one, being able to analyse non-rational models and models with unknown inputs (Villaverde *et al.*, 2019). (ii) *ProbObsTest* (Díaz *et al.*, 2023), which implements a version of Sedoglavic’s algorithm for analysing rational models, with two developments: it can analyse models with unknown polynomial inputs, and it can automatically transform models with logarithmic, trigonometric and exponential functions into rational models. (iii) *ORC-DF*, which was originally developed by Maes *et al.* (2019). In this study, we have evaluated the first two algorithms; we refer to these tools as STRIKE-GOLDD (FISPO) and STRIKE-GOLDD (ProbObsTest), respectively. We found that the ORC-DF implementation in STRIKE-GOLDD is less efficient than the implementation by Maes *et al.* (2019), and this one in turn is less efficient than RORC-DF, so we only considered the latter.

#### 2.5.9 StrikePy

StrikePy is a Python toolbox (available via pip) that analyses SLI (Rey Rostro and Villaverde, 2022). It implements the STRIKE-GOLDD (FISPO) algorithm, but it does not include other features present in that toolbox and is computationally less efficient than the Matlab implementation. On the other hand, at the moment of writing this article, it appears to be the only Python tool for analysing structural identifiability.

#### 2.5.10 RORC-DF

RORC-DF and ORC-DF are Matlab tools that follow a similar approach but have different applicability. ORC-DF (Observability Rank Criterion for systems with Direct Feedthrough) (Maes *et al.*, 2019) can analyse analytical models that are affine in the known and unknown inputs. The term *direct feedthrough* means that the outputs may be functions of the inputs. ORC-DF considers the unmeasured inputs and their time derivatives as additional states. RORC-DF (rational ORC-DF) (Shi and Chatzis, 2022) was the first extension of Sedoglavic’s algorithm to systems with unknown inputs. Unlike ORC-DF, RORC-DF does not require the system to be affine in the inputs, but it introduces the assumption of rational non-linearities. In RORC-DF, the observability matrix is composed by the coefficients of the power series expansion of the output functions, obtained with Newton’s iteration. Similar to ObservabilityTest, EAR and ProbObsTest, computations are carried out using random numerical realizations of the symbolic variables and applying modular operations to reduce the computational burden. RORC-DF is more efficient than ORC-DF.

## 3 Results and discussion

To benchmark the tools, we assembled a large and diverse collection of problems from the systems biology literature. Our collection consists of 25 problems created from 21 basic models, which are listed in Table 2 along with their references and dimensions (numbers of states, parameters, outputs and inputs). The collection includes rational and non-rational models, as well as models with and without inputs. For some of the latter, we consider both the known and the unknown input case. The smallest models that we consider have a few parameters and states, while the largest have tens of them. While larger models with hundreds or even thousands of parameters are increasingly common in systems biology, currently existing tools are not capable of analysing them. In our assessment, we consider several criteria, which are discussed in the following subsections. Table 3 summarizes the results of our analyses. All analyses were performed in a computer with 16GB RAM and 12-Core 3.80 GHz CPU.

**Table 2.** List of benchmark models and their main features

Short name	Reference	States	Param.	Kn-in	Unk-in	Outputs	Rational
C2Ma	Villaverde *et al.* (2019)	2	4	1		1	✓
C2Mb	Villaverde *et al.* (2019)	2	4			1	✓
C2Mc	Villaverde *et al.* (2019)	2	4		1	1	✓
Competition	Coleman and Gomatam (1972)	2	6			1	
HIV 1a	Perelson and Nelson (1999)	3	5	1		2	✓
HIV 1b	Perelson and Nelson (1999)	3	5		1	2	✓
HIV 2	Perelson and Nelson (1999)	4	10			2	✓
HIV 3	Wodarz and Nowak (1999)	5	10			2	✓
NFkB 1	Lipniacki *et al.* (2004)	15	29			6	✓
NFkB 2	Lipniacki *et al.* (2004)	15	6	1		6	✓
Phosphorylation	Conradi and Shiu (2018)	6	6			2	✓
PK 1	Merkt *et al.* (2015)	4	9			2	✓
PK 2	Verdiere *et al.* (2005)	4	9			1	✓
Ruminal lipolysis	Moate *et al.* (2008)	5	4			3	✓
Tumor	Thomas *et al.* (1989)	5	5			1	✓
MAPK	Nguyen *et al.* (2015)	3	14			3	
*A.thaliana*	Locke *et al.* (2005)	7	29	1		2	
Toggle switch a	Lugagne *et al.* (2017)	2	10	2		2	
Toggle switch b	Lugagne *et al.* (2017)	2	10		2	2	
JAK-STAT 1	Raia *et al.* (2011)	10	23	1		8	✓
JAK-STAT 2	Bachmann *et al.* (2011)	25	24			14	✓
β IG	Topp *et al.* (2000)	3	5	1		1	✓
SIRS with forcing	Weber *et al.* (2001)	5	13	1		2	✓
Cholera	Lee *et al.* (2017)	4	7			2	✓
Gene p53	Distefano (2015)	4	25	1		4	✓

*Note*: The columns display a short name for the model, its original publication, the number of its states, parameters (‘param.’), known inputs (‘Kn-in’), unknown inputs (‘Unk-in’), measured outputs and whether it is rational (indicated by a tick symbol) or not.

**Table 3.** Summary of results obtained with local (STRIKE-GOLDD, StrikePy, ObservabilityTest, RORC-DF and EAR) and global (DAISY, GenSII, SIAN, COMBOS and StructuralIdentifiability) identifiability tools

	STRIKE-GOLDD (FISPO)	STRIKE-GOLDD (ProbObsTest)	StrikePy	Observability test	RORC-DF	EAR	DAISY	GenSSI	SIAN (Maple)	SIAN (Julia)	SIAN (web)	COMBOS	Structural identifiability
C2M	0.63	1.58	2.77	0.03	3.40	0.05	0.34	2.81	0.358	10.27	0.376	0.31	33.91
C2Mb	1.17	1.72	7.97	0.05	4.58	0.11	0.24*	7.08	0.28	10.27	0.623	0.98	33.71
C2Mc	12.55	4.30	37.90	N/A	16.21	N/A	N/A	N/A	N/A	N/A	N/A	N/A	N/A
Compet.	1696.29	7.42*	OoT	N/A	N/A	N/A	N/A	OoM	N/A	N/A	N/A	N/A	N/A
HIV 1a	0.74	3.96	0.52	0.09	7.74	0.19	0.13*	0.73	0.80	12.09	0.821	0.83	32.02
HIV 1b	2.23${}^{⋄}$	8.65${}^{⋄}$	24.31${}^{⋄}$	N/A	11.27${}^{⋄}$	N/A	N/A	N/A	N/A	N/A	N/A	N/A	N/A
HIV 2	29.79	10.34	1685.76	0.22	40.59	0.6095	0.448*	966.66*	6.687	10.63	5.18	36.23	31.60
HIV 3	8528.00	12.76	OoT	0.20	36.42	1.25	6.66	751.74*	31.34	14.32	24.67	Error	32.78
NFkB1	33 345.00	304.40	OoT	8.42	11 666.91	24.37	OoT	6722.98*	3867.02	OoM	Error	Error	OoM
NFkB2	1007.00	329.83	OoT	3.14	1138.97	6.26	OoT	660.36	3690.70	244.27	Error	Error	OoM
Phospho.	1.87	13.41	32.40	0.16	28.05	0.91	19.43	974.61	5.23	13.64	4.06	Error	35.02
PK 1	2.69	6.41	198.96	0.14	34.00	0.58	0.31	14.58*	1.48	12.26	1.49	5.72*	34.39
PK 2	OoM	16.41	OoT	0.14	14.87	0.58	OoT	5082.68*	OoM	OoM	Error	Error	84.86
Ruminal	0.74	17.07	22.83	0.13	12.95	0.38	0.12	1.46	0.95	14.12	1.25	220.00	34.86
Tumor	24.86	8.66	636.55	0.17	140.13	1.00	8.91	1433.22	940.55	404.96	3.06	6735.38	34.87
MAPK	94.219	N/A	OoT	N/A	N/A	N/A	N/A	27.80	N/A	N/A	N/A	Error	N/A
*A.thal*	167 769.33${}^{⋄}$	N/A	N/A	N/A	N/A	N/A	N/A	6356.60${}^{⋄}$	N/A	N/A	N/A	N/A	N/A
TS a	62.85	N/A	OoT	N/A	N/A	N/A	N/A	N/A	N/A	N/A	N/A	N/A	N/A
TS b	29.497	N/A	OoT	N/A	N/A	N/A	N/A	N/A	N/A	N/A	N/A	N/A	N/A
JS 1	31.26	203.26*	OoT	2.00	1723.97	4.92*	OoT	23 284.00*	246.48	40.90	Error	Error	62.55
JS 2	146 450.00${}^{⋄}$	2318.46${}^{⋄}$	OoT	35.74${}^{⋄}$	86 333.30${}^{⋄}$	Error	OoT	N/A	115 200.00_*L*_${}^{⋄}$	OoM	Error	Error	37.21_*L*_${}^{⋄}$
β IG	2059.89	16.10	OoT	0.08	9.60	0.7663	0.09*	16 999.00*	6.54	11.33	3.38	Error	31.65
SIRS	87.98	9.20	2836.98	0.13	31.05	0.54	OoT	648.26*	10.94	12.13	6.57	Error	41.56
Cholera	162.26	8.02	210.39	0.11	14.02	0.46	OoT	361.08	380.67	56.52	261.72	Error	34.72
p53	308.05	112.53	29 193.25	0.34	45.35	3.32	0.792	OoM	221.73	93 31.52	411.25	1339.38	33.15

*Note*: The table entries display the runtimes for each case study in seconds. An asterisk (*) next to a value denotes that the result is thought to be wrong, while a diamond (${}^{⋄}$) denotes that the correctness of the result is unclear. OoM, Out of Memory error; OoT, Out of Time error (the computations were aborted if they surpassed a 48 h limit); Error, an unspecified error. A subscript 'L' indicates that the method could only produce local results.

### 3.1 Software accessibility and usability

Most toolboxes are freely available on a website, except EAR and DAISY, which are available upon request by email. All the toolboxes provide either a README file or a user manual, or both. As for debugging, some programming environments such as Matlab, Julia and Maple provide detailed reports of the problems encountered when executing a code. Other environments, namely Reduce, Mathematica and the COMBOS WebApp, do not specify the cause of the problem.

### 3.2 Possibility of performing a given analysis

#### 3.2.1 Types of models

The most common limitation regards the analysis of non-rational models, which can only be performed by STRIKE-GOLDD (FISPO), StrikePy and GenSSI. ProbObsTest, DAISY and COMBOS can deal with rational functions as long as they can be transformed into polynomial functions. In the case of functions with non-integer exponents (such as JAK-STAT 1 and βIG), their analysis with SIAN, StructuralIdentifiability, ObservabilityTest and EAR requires approximating their values to the closest integer. While, in general, this change should not alter the identifiability results, it can reduce computation times. Hence, if a model is modified in this way, it should also be modified when analysing it with other methods, in order to ensure a fair comparison. Another common limitation concerns models with unknown inputs. Only four methods can lead with this class of models, all of which use local approaches: RORC-DF, STRIKE-GOLDD (FISPO and ProbObsTest) and StrikePy. However, only RORC-DF can deal with non-polynomial unknown inputs.

#### 3.2.2 Types of analyses

Some methods can only determine structural local identifiability, while others can also analyse global identifiability. Furthermore, some tools (StrikePy and RORC-DF) provide only identifiability and observability results, while others also search for symmetries, identifiable parameter combinations or reparameterizations. Table 1 lists the main features of each tool. Some global tools such as SIAN, ObservabilityTest, DAISY, COMBOS and GenSSI provide information about the number of local solutions. Some local tools such as EAR, STRIKE-GOLDD and ObservabilityTest assist in finding symmetries and model reparameterizations.

#### 3.2.3 Computational feasibility

The feasibility of the analysis in practice must also be considered: even if a tool can analyse a given model in principle, it may not be able to do so due to computational limitations. This is reflected in the number of errors shown in Table 3. There are three types of error in this table, denoted by ‘OoM’, ‘OoT’ or ‘Error’. The ‘OoM’ acronym refers to the cases that yielded an ‘Out of memory’ message in Matlab or a ‘Connection to the kernel was lost’ message in Maple. We set an execution time limit of 48 h; if an analysis surpassed this limit, it was aborted and the result was reported as an ‘Out of time’ (OoT) error in the table. In other cases, the analyses ended prematurely (i.e. the tool was unable to analyse the model due to implementation limitations) without reporting any error message. These cases are indicated as an entry ‘Error’ in the table. Overall, the most computationally limited tools were StrikePy and COMBOS, whose limitations may be due to their implementations in Python and as a web app, respectively. DAISY yielded less errors than StrikePy and COMBOS, but it struggled with medium-sized models.

### 3.3 Results

#### 3.3.1 Correctness

Even when a tool has produced results for a given model, they may not always be correct. For some models, we found discrepancies among the results of several tools. In such cases, there was typically a clear consensus among methods, with only one or two methods that disagree with the common solution; in this case, we assumed that the consensus solution is the true one, and we marked the wrong solutions with an asterisk (*) in Table 3. However, in three cases (HIV 1b, JAK-STAT 2 and Arabidopsis *thaliana*) there was not a clear majority; in these cases, we did not make any assumptions about correctness, and we wrote a diamond (${}^{⋄}$) next to all results in the tables. Under these assumptions, we found that five methods did not produce any wrong result: SIAN, StructuralIdentifiability, ObservabilityTest, RORC-DF and STRIKE-GOLDD (FISPO). Two algorithms, EAR and STRIKE-GOLDD (ProbObsTest), yielded wrong results for JAK-STAT 1. We have realized that this result depends on the choice of prime number used by these methods to specialize the variables on random numbers; if we select the same prime number we obtain the same result. Additionally, STRIKE-GOLDD (ProbObsTest) yielded a wrong result for the Competition model, which could only be analysed with this method and with STRIKE-GOLDD (FISPO). This case study illustrates the following issue: due to the presence of logarithmic terms, methods such as ProbObsTest must transform the model into polynomial form in order to analyse it; however, the transformed model does not necessarily preserve the properties of the original model. Two other tools, DAISY and GenSSI, produced wrong results for a number of case studies. We contacted the authors of these tools to rule out the possibility of having obtained spurious results.

#### 3.3.2 Computational performance

Even when two tools agree on the result, their computational costs may be very different. Table 3 shows CPU times, which we have used as the main measure of this criterion. They depend on the programming environment and the algorithm. Clearly, the fastest algorithm in our tests was the one by Sedoglavic (2002), which is implemented with some variations in four toolboxes—ObservabilityTest, EAR, RORC-DF and STRIKE-GOLDD (ProbObsTest)—programmed in three different languages—Maple, Mathematica and Matlab. The fastest implementation was the Maple one, followed by the Mathematica one. Those three tools are restricted to structural *local* identifiability analysis (global tools are usually slower). Among the remaining local tools, the next two in terms of computational efficiency were STRIKE-GOLDD (FISPO) and RORC-DF. The slowest tool of all was StrikePy. Among *global* tools, GenSSI yielded the largest CPU times; DAISY was on average faster than GenSSI, although it managed to complete the analysis of fewer models (probably due to the 48-h limit that we imposed to the calculations). We found a similar but even more pronounced effect for COMBOS. In comparison, SIAN and StructuralIdentifiability performed very well. The computation times of StructuralIdentifiability were remarkably similar for most models, regardless of their size. We tested three implementations of SIAN, in Maple, Julia and as a web app. The Maple implementation was faster than the Julia one for smaller models and slower for larger models. Julia uses Just-in-time compilation, where each function is compiled the first time it is called. Therefore, the computation times in Table 3 count this compilation time together with loading the package, which may be about 20–30 s. Lastly, the SIAN web app has a runtime limitation due to the Maple server, which prevents it from analysing larger models (shown as ‘Error’ in Table 1). A workaround to this issue could be to use the Maple Player to run the app offline.

## 4 Conclusions

### 4.1 General guidelines

The process of selecting a tool must begin with the *type of model* that is going to be analysed. If it is a rational model without unknown inputs (a common situation in systems biology), all methods can be applied. However, for other model types, the choice of applicable methods is reduced, as can be seen in Table 1.

Second, the user must decide whether to assess *global* (*SGI*) *or local* (*SLI*) structural identifiability, if both approaches are applicable to the model. SGI implies SLI but the opposite is not true; while it is often the case that an SLI model is indeed SGI, some counter-examples have been reported. The extent to which the distinction between local and global identifiability is relevant in biological applications is worthy of further investigation. If it is not necessary to assess SGI, it may be convenient to resort to SLI methods, since they are usually computationally cheaper than SGI methods. When the SGI analysis of a model is too computationally demanding, a possible course of action could be to perform an SLI analysis first and use the results to search for reparameterizations of the model. Then, the SGI analysis of the reparameterized model may be attempted.

Another factor is the *software environment*. We have benchmarked tools written in six different programming languages, as well as some web-based applications that do not require the installation of specific software. While the array of available methods is reasonably large, for a given language the number of possible choices is usually restricted to two or at most three, and sometimes only one. Thus, the (in)convenience of reimplementing the model in a different language needs to be taken into account when choosing a software tool. This is especially important if the structural identifiability analysis is performed as part of a larger computational pipeline for model building and exploitation, which is a typical scenario. In this case, it is desirable to be able to perform all analyses within the same software environment. It should also be taken into account that some of the environments are proprietary software (Matlab, Mathematica and Maple), and therefore not available to every user.

Finally, some tools provide *additional features* that can assist in reformulating an unidentifiable model. Such features include the search for symmetries in the model equations, identifiable parameter combinations and identifiable model reparameterizations.

After considering the aforementioned factors, there may be several tools that meet the requirements for the problem at hand. In this case, the user may choose the one with the lowest *computational cost*. As our results have shown, computation times can vary greatly from one tool to another.

### 4.2 Recommendations

From the above discussion, it is apparent that the choice of the most appropriate tool is strongly problem dependent. While every tool has its particular merits, not all of them are equally useful. Hence, we would like to provide some final recommendations, which can be summarized as follows.

Within the tools that analyse structural *global* identifiability, there is a clear distinction between the more recent ones and the older ones. DAISY was the first tool of its kind to be made publicly available; however, the array of models that it is capable of analysing is currently smaller than that of other tools. The next one to appear, COMBOS, was a welcome innovation at the time of its release, thanks to its web app implementation; however, it exhibits similar or worse limitations as DAISY. The two most recent methods, SIAN and StructuralIdentifiability, do not share the limitations of the oldest ones. GenSSI lies somewhere in the middle of both groups. In summary, we recommend using either SIAN (Maple) or StructuralIdentifiability (Julia) for analysing structural global identifiability. The choice between them can boil down to a matter of software environment.

The tools that analyse structural *local* identifiability do not exhibit the same differences in performance between older and newer implementations. We can classify them into two groups, depending on whether they use some version of Sedoglavic’s algorithm—ObservabilityTest, EAR, STRIKE-GOLDD (ProbObsTest) and RORC-DF—or not—STRIKE-GOLDD (FISPO) and StrikePy. The first group yields faster calculations than the second one, but it cannot analyse non-rational models. For the analysis of *rational* models, we recommend, in order of computational efficiency, (i) ObservabilityTest, which is by far the fastest tool; (ii) EAR; (iii) STRIKE-GOLDD (ProbObsTest) or RORC-DF. Naturally, the final decision depends on the access to Maple, Mathematica and Matlab environments. For the analysis of non-rational models, STRIKE-GOLDD (FISPO) is in some cases the only available option. StrikePy does not outperform other tools and, given its limitations, it should be avoided unless it is necessary to perform the analysis in Python.

### 4.3 Directions for future research

As our results have shown, recent developments have yielded considerable advances in the available tools for structural identifiability analysis. However, further improvements are still needed to facilitate the analysis of more models, as they tend to become larger and more complex. In this regard, a promising line of work would be to implement more features in the Julia programming language, due to its computational efficiency. It should also be noted that all the tools considered in this article analyse ODE models. While they are the most common ones in systems biology, other types of models are also useful, such as those with partial differential equations or stochastic dynamics. The development of tools for their analysis would greatly broaden the applicability of structural identifiability analysis.

## Data Availability

The data underlying this article are available in GitHub, at https://github.com/Xabo-RB/Benchmarking_files.
